# Health-Related Quality of Life among Patients with Hepatitis C Virus Infection: A Cross-Sectional Study in Jianping County of Liaoning Province, China

**DOI:** 10.1155/2020/6716103

**Published:** 2020-01-07

**Authors:** Hairui Zhang, Ran Ren, Jinlin Liu, Ying Mao, Guowei Pan, Ke Men, Li Ma

**Affiliations:** ^1^Institute for Research on Health Information and Technology, School of Public Health, Xi'an Medical University, Xi'an, Shaanxi 710021, China; ^2^School of Public Health, Dalian Medical University, Dalian, Liaoning 116044, China; ^3^School of Public Policy and Administration, Xi'an Jiaotong University, Xi'an, Shaanxi 710049, China; ^4^Center for Disease Prevention and Control of Liaoning Province, Shenyang, Liaoning 110001, China

## Abstract

**Background:**

Patients with chronic liver disease (CLD) have extrahepatic manifestations and impaired health-related quality of life (HRQOL), and hepatitis C virus (HCV) infection is a leading cause of CLD, cirrhosis, and hepatocellular carcinoma (HCC). This study is aimed at assessing HRQOL in patients with HCV infection in the rural areas and identifying factors associated with impairment of HRQOL.

**Methods:**

A cross-sectional study was conducted in a county of Liaoning Province in northeast China. HRQOL of patients with HCV infection was assessed using the chronic liver disease questionnaire (CLDQ) and EuroQol-5 dimensions (EQ-5D). Data were transformed to score comparisons of six major CLDQ domains, EQ index, and visual analog scale (VAS).

**Results:**

A total of 397 (93.4%) subjects, including 67 healthy subjects (HSs), 314 patients with chronic hepatitis C (CHC), and 16 patients with liver cirrhosis (LC) completed the study. The overall quartile CLDQ scores for HSs, patients with CHC, and patients with LC were 6.4 (6.0, 6.7), 5.8 (4.6, 6.4), and 4.1 (3.0, 6.0), respectively. The quartile scores of EQ index for the three groups were 1.0 (1.0, 1.0), 1.0 (0.8, 1.0), and 0.9 (0.6, 1.0), respectively. The median scores of EQ VAS for the three groups were 85.0, 60.0, and 60.0, respectively. Female sex, patients with family history of hepatitis, other comorbid chronic diseases, drinking, and disease duration ≥ 10 years were associated with significant improvement in overall CLDQ scores, and family history of hepatitis and other comorbid chronic diseases were considered predictive factors for EQ index and VAS, respectively.

**Conclusions:**

Compared with HSs, HCV infection had a greater negative impact on HRQOL in patients with CHC and LC. The significant factors associated with HRQOL include female sex, patients with a family history of hepatitis, other comorbid chronic diseases, drinking, and disease duration ≥ 10 years. Patients with HCV infection in the rural areas should be paid careful attention regarding their HRQOL with proper health education and disease management.

## 1. Introduction

Viral hepatitis ranks first among infectious diseases, and the chronic hepatitis C (CHC) has currently been considered the “invisible killer” of chronic hepatitis in China [1]. According to the recent data released by the World Health Organization, the global prevalence of HCV is 3%, and approximately 170 million people are infected with HCV, with the annual incidence of new cases being approximately 35,000 [2, 3]. Of these patients, 20%–30% progress into liver cirrhosis (LC) or hepatocellular carcinoma (HCC) [4]. However, the public, specifically individuals living in the rural areas, has insufficient knowledge on the route of transmission and the corresponding preventive measures for CHC.

Health-related quality of life (HRQOL), as an objective instrument for evaluating patients' physical, psychological, and social adaptation and other indicators, has become significantly favorable to clinical doctors and health managers [5]. Most studies revealed that evaluating HRQOL has become important in public health and clinical research because it was considered the gold standard to report patient's experiences with illness and treatment [6]. However, China has a large number of patients with hepatitis, but less attention has been paid to the HRQOL of patients with HCV infection. Due to insufficient knowledge on health care and effective medical assistance, the HRQOL of patients in the rural areas is more severe than that in the urban areas and hence should be paid careful attention.

Previous international studies have reported a consistent and marked reduction in HRQOL in patients with chronic hepatitis [7]. Although few studies that assessed the HRQOL in different sociodemographic characteristics in patients with HCV were conducted in China, the associations between each component of the HRQOL and other patient factors remain unclear.

Thus, this study is aimed at evaluating HRQOL outcomes of patients with HCV infection in rural areas and identifying factors associated with impairment of HRQOL.

## 2. Materials and Methods

### 2.1. Patients

Since August 2014, to analyze the comprehensive prevention and treatment of CLD in Liaoning Province, the National Ministry of Science and Technology of China commissioned the Center for Disease Control and Prevention of Liaoning Province to complete the research. This study conducted an epidemiological screening in CLD research from August 2014 to June 2015. As a cross-sectional study, the convenient sampling method was used, subjects volunteered to participate in the survey, and the information was consecutively collected from patients with CHC, patients with LC, and the HSs. The diagnostic criteria of the disease were established following the standards in the guideline of prevention and therapy of hepatitis C, which was issued by the Hepatology Branch, Infectious Disease & Parasitologic Diseases Branch, Chinese Medical Association [8]. All patients were aged greater than 18 years who were infected with hepatitis C greater than 6 months. Patients were excluded if they had severe cognitive impairment, were coinfected with other types of hepatitis virus (hepatitis A virus, hepatitis B virus, hepatitis D virus, and hepatitis E virus), or refused to provide written informed consent. HSs were enrolled, and all subjects provided written informed consent for inclusion in the study.

Face-to-face interviews were conducted by well-trained interviewers and noninductive language was used for investigation. In order to protect the information of subjects, researchers cannot identify the information of each individual. Subjects' sociodemographic characteristics, including age, gender, marital status (married or not married), education level (no schooling and primary, secondary, and tertiary), comorbidity chronic illness, family history of hepatitis, and disease duration, were collected. Meanwhile, subjects' HRQOL was measured using the CLDQ and EQ-5D. The questionnaire will be excluded if the missing data exceeds 20% and above.

### 2.2. Questionnaires

The CLDQ is a standardized disease-specific HRQOL measurement for liver disease that is developed by Younossi et al. [9]. The questionnaire consists of 29 items that are divided into the following six domains: abdominal symptoms (AS), fatigue (FA), systemic symptoms (SS), activity (AC), emotional function (EF), and worry (WO). Each item is rated on a 7-point Likert scale ranging from 1 (all the time) to 7 (none of the time). The domain scores were calculated using the means of the items contained, and the overall score was the means of all item scores. In 2003, the CLDQ has been formally translated from the original version into Chinese version and was approved to be a reliable and valid questionnaire in assessing HRQOL in patients with CLDs [10].

EQ-5D is one of the most widely used generic scales that was presented by the EuroQol Group [11]. It consists of the following two parts: the EQ-5D descriptive system and the EQ visual analog scale (EQ VAS). The EQ-5D descriptive system comprises the following five domains: mobility, self-care, usual activities, pain/discomfort, and anxiety/depression. Each domain has the following three levels: no problems, some problems, and severe problems, which can be transformed to a single measure called EQ index or utility value. It ranges from 0 (reference value assigned to death) to 1 (perfect health), with the possibility of negative values for health status considered worse than death [12]. EQ VAS asks people to assess their own health status through a vertical thermometer-like scale ranging from 0 to 100, where 0 reflects the worst imaginable health status and 100 the best imaginable health status. Since the EQ-5D was developed, it has been widely used in measuring the HRQOL of special and normal in many counties [13, 14]. It showed a good reliability compared to other utility measures for both self and proxy ratings in people, making it therefore one of the most common instruments in evaluating HRQOL [15].

### 2.3. Statistical Analysis

Descriptive analysis for continuous variables was performed with mean ± standard deviation for normally distributed data and interquartile range and median for nonnormally distributed data. Categorical variables were described by counts and related percentages. Pearson's chi-square test and Fisher's exact test were used to compare the distribution of sociodemographic variables between all patients and the healthy subjects. Mann-Whitney *U* test was performed to compare the HRQOL score of each domain of CLDQ and EQ-5D among the three groups. Associations between variables were measured using Spearman's correlation coefficient to examine the association between each domain of CLDQ and EQ-5D. Additionally, multivariate linear regression was performed to estimate independent association of each variable with the score of overall, EQ index, and VAS. All tests were two-sided with a significance level < 0.05. All data were double-typed with Epidata3.2 by two different persons to avoid data errors, and SPSS, version 16.0 (SPSS Inc., Chicago, IL, USA) was used to conduct all the analysis.

## 3. Results

### 3.1. Subject Characteristics

All subjects were from rural areas in Jianping County, and a total of 425 subjects participated in the study, of which 28 were excluded because they did not complete the questionnaire. A total of 397 (93.4%) subjects, which included 67 HSs, 314 patients with CHC, and 16 patients with LC, completed the study, and the mean ages were 51.5 ± 9.5, 55.2 ± 8.2, and 60.4 ± 7.4 years, respectively. Greater than two-fifths (42.3%) were aged from 50 to 59 years old. A total of 67.5% of the subjects were female. Regarding marital status, most of the subjects (96.0%) were married and lived with their families; subsequently, the remaining subjects had no spouse (divorced, dead, or not married). As regards disease duration, 58.8% of the patients (CHC and LC) had disease duration greater than 5 years. Information about subject's sociodemographic characteristics is summarized in Table 1.

### 3.2. The HRQOL Score of CLDQ and EQ-5D

We calculated the CLDQ and EQ-5D scores for every domain according to the subject's specific response using the computational formula that is commonly used in Table 2. Except for patients with LC who had a normal distribution of scores in all domains, the scores of the other groups in each domain showed nonnormal distribution. The overall quartile CLDQ scores for HSs, patients with CHC, and patients with LC were 6.4 (6.0, 6.7), 5.8 (4.6, 6.4), and 4.1 (3.0, 6.0), respectively. Using the EQ index, subjects scored 1.0 for HSs, 1.0 for CHC, and 0.9 for LC. The median scores of EQ VAS for the three groups were 85.0, 60.0, and 60.0, respectively. Using the Mann-Whitney *U* test, all the domain CLDQ and EQ-5D scores were significantly lower in patients with CHC than that in HSs, and similar results were observed in patients with LC and HSs. The median scores of the patients with CHC were significantly higher for FA, SS, AC, EF, WO, and overall CLDQ, except for AS, WO, and EQ-5D, compared to that of patients with LC.

### 3.3. The Correlation between CLDQ and ED-5D

All CLDQ domains were significantly associated with all the scores of EQ-5D subscales (*r* > 0.408, *P* < 0.001); the associations between the overall score and each domain score using the EQ index and EQ VAS ranged from weak to moderate in Table 3. The overall score was highly associated with the six domains, and the associations between WO and other domains were the lowest. Compared to the EQ index, EQ VAS had a week association with the overall and each domain score of CLDQ. Compared to the six domains, the strongest association was observed between the overall score of the CLDQ and all EQ-5D domains, followed by the associations between the domains of SS and EF.

### 3.4. Independent Predictors of HRQL Scores

Multivariate linear regression analysis predicted the overall CLDQ scores, EQ index, and VAS.  Table 4 shows the beta coefficients (95% confidence interval (CI)) obtained from each independent variable, which accounted for in the regression model. Regression analysis showed that female sex (*β* = 0.435; 95% CI, 0.084 to 0.786), patients with family history of hepatitis (*β* = −0.406; 95% CI, -0.723 to -0.088), other comorbid chronic diseases (*β* = −0.300; 95% CI, -0.585 to -0.014), drinking (*β* = −0.347; 95% CI, -0.677 to -0.017), and disease duration ≥10 years (*β* = −0.518; 95% CI, -0.826 to -0.211) were predictive factors of overall CLDQ scores.

Notably, independent factors for EQ index and VAS were not similar to that of overall CLDQ scores. As shown in Table 4, patients with family history of hepatitis (*β* = −0.056; 95% CI, -0.104 to -0.008) were a predictive factor for EQ index, and other comorbid chronic diseases (*β* = −5.857; 95% CI, -9.521 to -2.193) were predictive factor for EQ VAS.

## 4. Discussion

Using the EQ-5D and CLDQ simultaneously is the most promising approach to evaluate the HRQOL of patients with HCV infection in the present study. Compared to the generic scale, the disease specificity scale can more accurately express the specific clinical symptoms of patients [16]. We found that the HRQOL in patients with HCV infection was significantly impaired in all domains compared to that of HSs, signifying that chronic viral hepatitis is more commonly seen in the rural areas than the urban areas of China and that the chronic sequelae of chronic viral hepatitis, such as cirrhosis and HCC, may have greatly affected patients' health. We propose that patients with HCV infection in the rural areas should be paid careful attention regarding their HRQOL with proper health education and disease management [17].

A recent study, which was conducted using the Japanese version of the CLDQ among Japanese patients with chronic viral hepatitis, in Japan has reported that the CLDQ scores of these patients were lower compared to that of the present study except for the AC domain [18]. The median scores of the domains were as follows: AS with 6.3, FA with 5.6, SS with 5.6, AC with 5.7, EF with 5.7, WO with 6.3, and overall with 5.8. Scores of all CLDQ domains were significantly lower in patients with CHC than that in HSs, and similar results were observed in patients with LC and HSs in this study. According to the other studies, HCV elimination improves HRQOL in patients with CHC [19].

Some studies reported that psychiatric disorders, FA, and depression are common symptoms of HCV patients, which may constitute a major influencing factor for patients with low QOL [20, 21]. In the present study, the scores of FA and EF domains of CLDQ were relatively low among the six domains.

To the best of our knowledge, patients generally show significant WO about the harm caused by diseases, which can seriously affect their QOL [22, 23]. Interestingly, the WO domain had the highest score in all CLDQ domains among the three groups in our study, which was inconsistent with the other studies [24]. Since the study subjects came from rural areas, they had insufficient knowledge on the severity of the disease due to low educational level and little knowledge about viral hepatitis, so they are less worried and anxious [25]. These might explain the high HRQOL levels in CLDQ WO domain, and further efforts should be exerted regarding this issue to determine the causes of these differences in the future.

The results from generic questionnaire EQ-5D and disease-specific questionnaire CLDQ were in agreement with the fact that a marked decrease of QOL was observed in patients with HCV in this study. All EQ-5D domain scores were significantly lower in patients with HCV than that in HSs. The median EQ indices of patients with CHC and LC were 1.0 and 0.9, which were higher than that in Spain. EQ VAS of patients in the two groups was 60.0, which was lower than that in Spain [26].

Significant association between scores on EQ-5D subscales and CLDQ domains was observed. Overall CLDQ scores were highly associated with the six domains, and the associations between WO and other domains were the lowest. Compared to the EQ index, EQ VAS had a lower association with each domain of the CLDQ, and the results were consistent with the previous report [27]. The highly significant association between CLDQ and EQ-5D subscales provides further evidence for the convergent and discriminant validity of the CLDQ. Although CLDQ and EQ-5D do not define disease statuses, our study showed that these two methods could successfully represent the impaired HRQOL in patients with HCV.

Multivariate linear regression was performed to confirm the effect of variables on CLDQ and EQ-5D scores while controlling the influence of other variables. Regression analysis showed that female sex, patients with family history of hepatitis, other comorbid chronic diseases, drinking, and disease duration ≥ 10 years were the predictive factors of overall CLDQ scores. We found out that female sex had negative influence on HRQOL. Similarly, findings from a study in Thailand revealed that females have more health concerns and are more treatment-seekers than males [28]. Takahashi et al. reported that comorbid diseases such as primary biliary cholangitis may influence the HRQOL in patient with hepatitis [18]. This may be due to the fact that patients with CHC were originally unhealthy and had concurrent CLD; hence, the physiological function of the body would be significantly influenced. Regarding patients with family history of hepatitis, one possible explanation was that patients with the family history might be seriously affected by the family members both in physiological and in psychological aspects [19]. Compared to never drinker, regular drinker was also associated with worse CLDQ scores. Similarly, regarding disease duration, this factor was also negatively associated with CLDQ scores [18]. In this study, only patients with family history of hepatitis and other comorbid chronic diseases were predictive factors for EQ index and VAS, respectively. Despite the observed significant association between EQ-5D and CLDQ and the damage caused by liver disease in terms of specific performance of QOL, EQ-5D was not as sensitive as CLDQ [27].

## 5. Conclusions

Compared with HSs, HCV infection had a greater negative impact on HRQOL in patients with CHC and LC. The significant factors associated with HRQOL include female sex, patients with a family history of hepatitis, other comorbid chronic diseases, drinking, and disease duration ≥ 10 years. Patients with HCV infection in the rural areas should be paid careful attention regarding their HRQOL with proper health education and disease management.

## Figures and Tables

**Table 1:** Sociodemographic characteristics of all subjects.

Characteristics	HSs (*n* = 67)	CHC (*n* = 314)	LC (*n* = 16)	*P* value
Age (years)				<0.001
<50	33 (49.3)	86 (27.4)	1 (6.3)	
50-59	23 (34.3)	140 (44.6)	5 (31.2)	
≥60	11 (16.4)	88 (28.0)	10 (62.5)	
Gender				0.230
Female	43 (64.2)	217 (69.11)	8 (50.0)	
Male	24 (35.8)	97 (30.89)	8 (50.0)	
BMI (kg/m^2^)				0.251
<18.5	4 (6.0)	11 (3.5)	1 (6.3)	
18.5-23.9	46 (68.7)	188 (59.9)	11 (68.7)	
≥24	17 (25.3)	115 (36.6)	4 (25.0)	
Marital status				0.332
Married	63 (94.0)	303 (96.50)	15 (93.7)	
No spouse^1^	4 (6.0)	11 (3.50)	1 (6.3)	
Education status				0.001
Primary school and below	24 (35.8)	179 (57.0)	13 (81.3)	
Junior school	30 (44.8)	114 (36.3)	3 (18.7)	
Senior school and above	13 (19.4)	21 (6.7)	0 (0.0)	
Annual family income (Yuan)				<0.001
<10000	7 (10.4)	70 (22.3)	5 (31.2)	
10000-19999	13 (19.4)	107 (34.1)	7 (43.8)	
20000-29999	11 (16.4)	70 (22.3)	2 (12.5)	
≥30000	36 (53.7)	67 (21.3)	2 (12.5)	
Other comorbid chronic diseases				<0.001
Yes	1 (1.5)	116 (36.9)	3 (18.7)	
No	66 (98.5)	198 (63.1)	13 (81.3)	
Family history of hepatitis				<0.001
Yes	2 (3.0)	76 (24.2)	3 (18.7)	
No	65 (97.0)	238 (75.8)	13 (81.3)	
Smoking status				0.922
Current smoker	33 (49.3)	150 (47.8)	7 (43.7)	
Nonsmoker	34 (50.7)	164 (52.2)	9 (56.3)	
Drinking status				0.004
Current drinker	33 (49.3)	90 (28.7)	4 (25.0)	
Nondrinker	34 (50.7)	224 (71.3)	12 (75.0)	
Disease duration (years)				0.806
<5	/	128 (40.8)	8 (50.0)	
5-9	/	80 (25.5)	3 (18.8)	
≥10	/	106 (33.7)	5 (31.2)	

The data are expressed as counts (percent); BMI: body mass index calculated as weight/height^2^ (kg/m^2^); CHC: chronic hepatitis C; LC: liver cirrhosis; HSs: healthy subjects. ^1^No spouse aspect includes single, divorced, and widowed.

**Table 2:** CLDQ and EQ-5D scores among patients with CHC, LC, and healthy subjects.

Domain	HSs	CHC	LC	*P* ^∗^	*P* ^∗∗^	*P* ^∗∗∗^
CLDQ						
Abdominal symptoms	6.7 (6.0-7.0)	6.3 (5.0-7.0)	5.0 (3.0-6.9)	0.006	0.004	0.053
Fatigue	6.2 (5.4-6.6)	5.6 (4.0-6.6)	3.9 (2.1-5.9)	0.012	0.001	0.019
Systemic symptoms	6.2 (5.8-6.8)	5.6 (4.4-6.2)	3.4 (2.4-5.4)	<0.001	<0.001	0.001
Activity	6.7 (6.3-7.0)	5.7 (4.6-7.0)	4.0 (3.1-6.6)	<0.001	0.001	0.035
Emotional function	6.5 (5.8-6.9)	5.7 (4.5-6.5)	3.9 (2.9-5.9)	<0.001	<0.001	0.010
Worry	7.0 (6.6-6.0)	6.3 (4.6-7.0)	5.2 (3.5-7.0)	<0.001	0.003	0.281
Overall	6.4 (6.0-6.7)	5.8 (4.6-6.4)	4.1 (3.0-6.0)	<0.001	<0.001	0.008
EQ-5D						
Index	1.0 (1.0-1.0)	1.0 (0.8-1.0)	0.9 (0.6-1.0)	0.001	0.003	0.138
VAS	85.0 (80.0-90.0)	60.0 (70.0-80.0)	60.0 (50.0-80.0)	<0.001	0.001	0.318

The data are expressed as median (interquartile range). CHC: chronic hepatitis C; LC: liver cirrhosis; HSs: healthy subjects; CLDQ: chronic liver disease questionnaire; EQ-5D: EuroQol-5D; VAS: visual analogue scale. ^∗^HS vs. CHC, ^∗∗^HS vs. LC, and ^∗∗∗^CHC vs. LC.

**Table 3:** Spearman's correlation coefficients between CLDQ and EQ-5D domains.

Domains	AS	FA	SS	AC	EF	WO	Overall	Index	VAS
AS									
FA	0.608^∗^								
SS	0.634^∗^	0.690^∗^							
AC	0.598^∗^	0.734^∗^	0.651^∗^						
EF	0.618^∗^	0.731^∗^	0.685^∗^	0.696^∗^					
WO	0.469^∗^	0.558^∗^	0.524^∗^	0.552^∗^	0.662^∗^				
Overall	0.736^∗^	0.876^∗^	0.831^∗^	0.812^∗^	0.912^∗^	0.743^∗^			
Index	0.528^∗^	0.643^∗^	0.629^∗^	0.591^∗^	0.624^∗^	0.499^∗^	0.694^∗^		
VAS	0.408^∗^	0.523^∗^	0.535^∗^	0.489^∗^	0.580^∗^	0.479^∗^	0.618^∗^	0.508^∗^	

AS: abdominal symptoms; FA: fatigue; SS: systemic symptoms; AC: activity; EF: emotional function; WO: worry; VAS: visual analogue scale; CLDQ: chronic liver disease questionnaire; EQ-5D European quality of life questionnaire-5 dimensions. ^∗^Association is significant at the 0.01 level (2-tailed).

**Table 4:** Multivariate linear regression for predictive factors on the overall CLDQ scores and EQ-5D in patients with HCV infection (CHC and LC).

Variables	Overall CLDQ		EQ index		EQ VAS	
	*β* coefficient (95% CI)	*P* value	*β* coefficient (95% CI)	*P* value	*β* coefficient (95% CI)	*P* value
Gender						
Male	1		1		1	
Female	0.435 (0.084 to 0.786)	0.015	0.049 (-0.003 to 0.102)	0.066	3.696 (-0.807 to 8.199)	0.107
Marital status						
No spouse	1		1		1	
Married	-0.205 (-0.924 to 0.515)	0.576	-0.009 (-0.117 to 0.099)	0.869	-3.104 (-12.306 to 6.098)	0.507
Family history of hepatitis						
No	1		1		1	
Yes	-0.406 (-0.723 to -0.088)	0.013	-0.056 (-0.104 to -0.008)	0.022	-0.328 (-4.404 to 3.748)	0.874
Other comorbid chronic diseases						
No	1		1		1	
Yes	-0.300 (-0.585 to -0.014)	0.040	-0.030 (-0.073 to 0.013)	0.175	-5.857 (-9.521 to -2.193)	0.002
Smoking status						
No	1		1		1	
Yes	-0.099 (-0.436 to 0.238)	0.564	-0.015 (-0.066 to 0.036)	0.564	-0.344 (-4.672 to 3.983)	0.876
Drinking status (years)						
No	1		1		1	
Yes	-0.347 (-0.677 to -0.017)	0.040	-0.023 (-0.073 to 0.026)	0.355	-3.416 (-7.650 to 0.817)	0.113
Disease duration (years)						
<5	1		/		/	
≥10	-0.518 (-0.826 to -0.211)	0.001	/		/	

VAS: visual analogue scale; CLDQ: chronic liver disease questionnaire; EQ-5D: European quality of life questionnaire-5 dimensions.

## Data Availability

The data used to support the findings of this study are included within the article.
